# Diabetic Neuropathy and Axon Reflex-Mediated Neurogenic Vasodilatation in Type 1 Diabetes

**DOI:** 10.1371/journal.pone.0034807

**Published:** 2012-04-17

**Authors:** Maryam Nabavi Nouri, Ausma Ahmed, Vera Bril, Andrej Orszag, Eduardo Ng, Patti Nwe, Bruce A. Perkins

**Affiliations:** 1 Division of Endocrinology and Metabolism, Department of Medicine, University of Toronto, Toronto, Ontario, Canada; 2 Division of Neurology, Department of Medicine, University of Toronto, Toronto, Ontario, Canada; University of Padova, Italy

## Abstract

**Objective:**

Axon reflex-mediated neurogenic vasodilatation in response to cutaneous heating may reflect early, pre-clinical small fibre dysfunction. We aimed to evaluate the distribution of the vascular flare area measured by laser doppler imaging (“LDI_FLARE_ area”) in type 1 diabetes and in healthy volunteers.

**Research and Methods:**

Concurrent with clinical and electrophysiological examination to classify diabetic sensorimotor polyneuropathy (DSP), LDI_FLARE_ area (cm^2^) was determined in 89 type 1 diabetes subjects matched to 64 healthy volunteers. We examined the association and diagnostic performance of LDI with clinical and subclinical measures of DSP and its severity.

**Results:**

Compared to the 64 healthy volunteers, the 56 diabetes controls without DSP had significantly lower LDI_FLARE_ area (p = 0.006). The 33 diabetes cases with DSP had substantially lower LDI_FLARE_ area as compared to controls without DSP (p = 0.002). There was considerable overlap in LDI_FLARE_ area between all groups such that the ROC curve had an AUC of 0.72 and optimal sensitivity of 70% for the detection of clinical DSP. Use of a subclinical definition for DSP, according to subclinical sural nerve impairment, was associated with improved AUC of 0.75 and sensitivity of 79%. In multivariate analysis higher HbA1c and body mass index had independent associations with smaller LDI_FLARE_ area.

**Conclusions:**

Axon reflex-mediated neurogenic vasodilatation in response to cutaneous heating is a biomarker of early nerve dysfunction in DSP. Its independent association with glycemic exposure in diabetes subjects and both glycemic exposure and BMI in healthy volunteers highlights the existence of small-fibre dysfunction in the natural history of DSP.

## Introduction

The rising incidence of diabetes and its negative impact on quality of life highlights the urgent need to develop biomarkers of early nerve damage. Diabetic sensorimotor polyneuropathy (DSP) – the most common complication in type 1 diabetes – represents diffuse symmetrical and length dependent injury to peripheral nerves and predisposes patients to neuropathic pain, sensory and motor dysfunction, and other complications[1]–[6]. The underdiagnosis of neuropathy delays early management necessary to improve glycemic control and the prevention of neuropathy-related sequelae [4], [7].

The prevailing concept of the natural history of DSP is that early nerve damage begins in small thinly myelinated Aδ and unmyelinated C-type nerve fibres [8], [9]. The gold-standard method to evaluate morphological change in small nerve fibres has been the skin biopsy [10], however, this technique is limited by cost, invasiveness, provides no information about the function of nerve fibres and cannot be employed as a generalized screening test in all patients with type 1 diabetes. Currently, there is an urgent need for the application of a valid clinical screening test to detect early small fibre dysfunction.

Out of the current recommendations for detection of neuropathy, evaluation of the Semmes-Weinstein 10 g monofilament examination was shown to be a valid tool with good performance to identify DSP [11], [12]. Though much work has been accomplished to define the validity of simple sensory testing on physical examination for use in the clinic, there are limitations in specificity as pre-clinical, predictive markers to detect the onset of neuropathy [12]–[14].

As an alternative, axon reflex-mediated neurogenic vasodilatation (“LDI_FLARE_”) is directly related to nociceptive C-fibre function and has shown promise as a marker of DSP [15], [16]. A heat stimulus causes an increase in blood flow for several centimeters around the site of skin irritation through stimulation of peripheral C-fibre branches in surrounding skin. This activation causes conduction of a signal to branches of the same C-fibre nerve (antidromic transmission) triggering the release of vasoactive peptides to increase the diameter of cutaneous vessels. The neurogenic reflex depends on intact sensory innervations of the skin because the spread of the flare is largely ablated by the administration of local anesthesia, supporting that the LDI_FLARE_ area is neurogenic in nature [17]–[19].

As the first step to ultimately evaluate LDI as a screening test that can predict the future onset of DSP, we sought to determine the performance of LDI_FLARE_ area to identify the presence or absence of DSP in the cross-sectional baseline evaluation of an ongoing longitudinal cohort study of individuals with type 1 diabetes and healthy volunteers. We used a gold-standard definition for DSP based on nerve conduction studies but also sought to establish the diagnostic performance of a subclinical definition of DSP according to abnormality in individual nerve conduction parameters.

## Methods

Eighty-nine participants with type 1 diabetes were accrued from the Diabetes and Endocrinology Clinic and Diabetic Neuropathy Clinic at the Toronto General Hospital/University Health Network. Sixty four healthy volunteers, recruited through friends and family members of participants with diabetes and community advertisements, were part of a cohort study funded by the Juvenile Diabetic Research Foundation (operating grant No.17-2008-715). The main objective of this study was to identify the concurrent validity of laser doppler imaging (LDI) in the cross-sectional identification of DSP and its predictive validity in the longitudinal analysis of those without baseline neuropathy. The current report evaluates the cross-sectional data from examinations conducted between November 2008 and May 2010.

### Subject Selection and Evaluation

We aimed to include type 1 diabetes subjects with a spectrum of nerve damage, from lack of detectable nerve injury to severe DSP. This was accomplished by way of stratified accrual according to the Toronto Clinical Neuropathy Score (TCNS), a validated grading system to evaluate history and physical exam components that permitted tracking of the numbers of subjects likely to have absent, mild, moderate and severe neuropathy at the time of study accrual [13], [20]. Subjects were included if they had type 1 diabetes, were 18 years or older, provided informed consent and did not have neuropathy attributable to causes other than diabetes. The causes of neuropathy were excluded by detailed medical history, family history, history of toxin exposure, renal failure or presence of abnormal serum or urine protein electrophoresis. Healthy volunteers were selected according to age matching (by decade of life) and gender matching with the type 1 diabetes participants.

A comprehensive medical and neurologic evaluation of each participant and healthy volunteer involved a clinical neurological history and examination with a focus on lifestyle factors and comorbidities. Biochemical tests included glycated hemoglobin A1C, serum lipids and urinary albumin excretion.

### Assessment Of Axon–Reflex Mediated Neurogenic Vasodilatation In Response To Cutaneous Heating By The Laser Doppler Imaging Flare Technique (“LDI_FLARE_”)

The surface skin temperature of the dorsum of the foot was standardized to 32°C using a warm blanket. We subsequently used a standard skin-heating probe (Moore instruments Ltd, Axminster, UK), a 0.64 cm^2^ circular metal disc that was well-affixed to the skin above the first metatarsal area on the dorsum of the foot, that heated the skin to 44°C for 20 minutes. After probe removal the LDI_FLARE_ was measured by LDI using the moorLDI2™ (Moor Instruments Ltd, Axminster, UK) [15]. The laser head of the LDI apparatus was positioned at a fixed distance of 30 cm from the dorsum of the foot and scanned an area of 6 cm×6 cm (36 cm^2^). The LDI apparatus used a scanning doppler infrared laser beam with a wavelength of 785 nm, sufficient to penetrate skin to register the movement of blood cells in dermal capillaries. The 36 cm^2^ area represented a 256×256 pixel resolution with each pixel itself representing a measurement of the velocity of tissue blood flow. The total scanning time was less than five minutes per examination. The flare area (cm^2^) was calculated using Moor LDI software (version 3.11).

### Classification Of Diabetic Sensorimotor Polyneuropathy (DSP) Cases And Controls

Nerve conduction study (NCS) testing involved examination of unilateral dominant-side sural and peroneal nerves of the lower limb. Two sural parameters were studied, the sural nerve action potential amplitude and the sural nerve conduction velocity). Three peroneal nerve parameters were tested – the distal compound muscle action potential amplitude and the f-wave latency from ankle stimulation, and the conduction velocity from fibular head stimulation. The test was performed using the Counterpoint instrument (Natus Medical, San Carlos, CA) meeting the standards of the American Association for Neuromuscular and Electrodiagnostic Medicine and the Canadian Society of Clinical Neurophysiology.

DSP was established by clinical and electrophysiological criteria proposed by the American Association of Neurology, the American Academy of Electrodiagnostic Medicine and the American Academy of Physical Medicine and Rehabilitation. Based on this consensus, we defined electrophysiological abnormality according to two criteria; the first was the “England criteria” which included the presence of at least one abnormal nerve conduction parameter in both sural and peroneal nerve distributions in combination with the presence of more than one symptom (numbness, tingling, weakness, foot pain, or ataxia) or sign (abnormal knee or ankle reflexes, temperature, light touch, monofilament, or vibration sensation) [21]. We applied age- and height-adjusted criteria for sural and peroneal amplitudes and conduction velocities, which were scored as normal or abnormal according to laboratory testing values [22]. To meet criteria for DSP, the neurological manifestations must have been in keeping with a length-dependent symmetrical c pattern of onset and progression. The second case definition was for subclinical DSP, defined by the presence of one or more abnormalities in sural nerve amplitude or conduction velocity (“sural nerve criteria”).

**Table 1:** Clinical characteristics of the 64 healthy volunteers and of the 89 type 1 diabetes participants according to diabetic sensorimotor polyneuropathy status.

Baseline Clinical Characteristic	Healthy volunteers (n = 64)	Type 1 diabetes (n = 89)		ANOVA P-valuefor trend*
		Controls without DSP (n = 56)	Cases with DSP (n = 33)	
Female sex (%)	34 (53%)	29 (53%)	17 (52%)	0.99
Age (yr)	38.9 ± 17.6	34.9 ± 14.8	50.0 ± 14.3	0.0001
Diabetes Duration (yr)	–	17.6 ± 14.0	31.4 ± 13.5	<0.0001
Current/Recent Smoking, n(%)	13 (21%)	7 (13%)	7 (21%)	0.43
Body mass index (kg/m^2^)	24.7 ± 4.6	25.3 ± 4.4	28.9 ± 5.0	0.001
Height (m)	1.7 ± 0.1	1.7 ± 0.1	1.7 ± 0.1	0.14
Weight (kg)	71.7 ± 15.9	76.9 ± 15.2	85.5 ± 19.7	0.005
Systolic Blood Pressure (mmHg)	124 ± 14	125 ± 14	137 ± 17	0.0001
Diastolic Blood Pressure (mmHg)	76 ± 11	71 ± 8	73 ± 9	0.01
HbA1c (%)	5.5 ± 0.4	7.4 ± 1.3	8.7 ± 2.1	<0.0001
Total cholesterol (mmol/L)	4.9 ± 1.1	4.6 ± 0.8	4.6 ± 1.6	0.29
LDL cholesterol (mmol/L)	2.9 ± 0.8	2.5 ± 0.7	2.4 ± 1.1	0.01
HDL cholesterol (mmol/L)	1.5 ± 0.5	1.7 ± 0.4	1.6 ± 0.5	0.18
Triglycerides (mmol/L)	1.1 ± 0.6	0.9 ± 0.7	1.2 ± 0.9	0.16
Toronto Clinical Neuropathy Score, Median [IQR] †	0 [0,2]	2.5 [1.5, 6.0]	10 [7], [14]	<0.0001
Sural nerve amplitude potential (µV)	18 ± 8	11 ± 5	2 ± 2	<0.0001
Sural nerve conduction velocity (m/s)	51 ± 5	46 ± 4	40 ± 3	<0.0001
Peroneal nerve amplitude potential (mV)	6 ± 2	6 ± 2	2 ± 1	<0.0001
Peroneal nerve conduction velocity (m/s)	48 ± 3	43 ± 3	36 ± 5	<0.0001
LDI_FLARE_ Area (cm^2^) ‡	3.4 ± 1.9	2.4 ± 1.4	1.4 ± 0.6	<0.0001

Plus-minus values are means ± SD. [IQR] represents the interquartile range.

* P values for categorical variables were calculated with the χ2 test, and ANOVA was used for continuous variables.

† TCNS, Toronto clinical Neuropathy score. Scores of 0–5 are generally considered to represent low likelihood of DSP, 6–8 represents likelihood of mild neuropathy, 9–12 represent likelihood of moderate neuropathy, while 12–19 represent severe neuropathy.

‡ Axon-reflex mediated neurogenic vasodilatation by the laser doppler imaging flare method.

Among individuals with diabetes, subjects that did not meet “England criteria” for DSP were labeled as “controls”. To investigate a definition for subclinical nerve injury the diabetes control subjects were further divided into two groups based on “sural nerve criteria”: one group had normal sural nerve parameters and the other group included individuals with one or more abnormalities in sural nerve amplitude or conduction velocity. Subjects that met the “England criteria” for DSP were labeled as “cases” and designated into three groups by increasing neuropathy severity: mild, moderate and severe. The criteria for mild neuropathy were two or more abnormal nerve conduction parameters in the lower limb (sural and peroneal nerve distributions), and moderate and severe neuropathy were defined by four and five abnormal parameters, respectively.

### Statistical Analysis

Analysis was performed using SAS (version 9.2 for windows). Differences in clinical categorical variables between healthy volunteers and individuals with type 1 diabetes with and without DSP were assessed using the *χ^2^* test while continuous variables were assessed by ANOVA. The results are expressed as mean ± SD. Among the baseline variables (Table 1) the TCNS score was not normally distributed. As such, the data are presented as median and interquartile ranges. Dependent predictor variables for the univariate linear regression models with LDI_FLARE_ area were assessed by standard regression diagnostics. Receiver operating characteristic (ROC) curves were generated for LDI_FLARE_ area to determine the area under the curve (AUC) and for inspection of the optimal threshold value to detect DSP by the “England criteria” and the subclinical definition of neuropathy using “sural nerve criteria”.

## Results

We characterized 153 subjects in this cross-sectional analysis, consisting of 64 healthy volunteers, 56 individuals with type 1 diabetes without nerve damage and 33 individuals with type 1 diabetes and DSP. As shown in Table 1, individuals with type 1 diabetes and DSP were older, had longer diabetes duration, higher body mass index and weight, and higher systolic and diastolic blood pressure compared to both healthy volunteers and type 1 diabetes controls without DSP. Among the type 1 diabetes subjects, HbA1c was higher but LDL cholesterol was lower compared to healthy volunteers. The Toronto Clinical Neuropathy Score (TCNS), a clinical indicator of the severity of nerve injury, was also higher in subjects with type 1 diabetes compared to healthy volunteers. Consistent with this finding, sural and peroneal nerve amplitude potentials were lower and conduction velocities were slower in subjects with type 1 diabetes compared to healthy volunteers. The wide interquartile range of TCNS scores and proportionately large standard deviations for nerve conduction parameters in type 1 diabetes cases with DSP indicated a wide distribution of nerve injury. The LDI_FLARE_ area was smaller in subjects with type 1 diabetes compared to healthy volunteers (ANOVA p-value for trend, p<0.0001).

**Figure 1:**
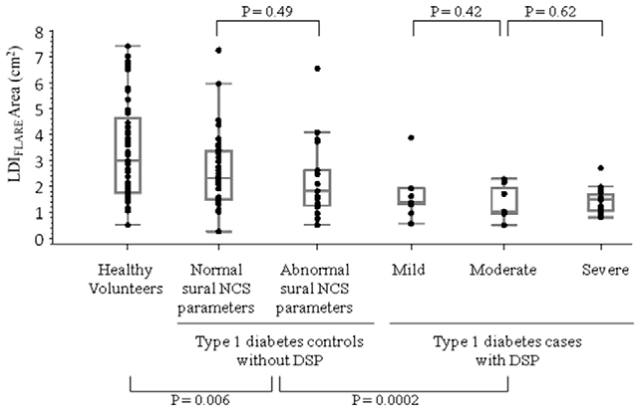
Box-And-Whisker Plots Demonstrating The Distribution Of LDI_FLARE_ Area In 64 Healthy Volunteers And 89 Type 1 Diabetes Subjects According To Neuropathy Status. Compared to the healthy volunteer group LDI_FLARE_ area was significantly smaller in subjects with type 1 diabetes without DSP (p = 0.006). Compared to subjects with type 1 diabetes controls without DSP, LDI_FLARE_ area was smaller in cases with DSP (p = 0.0.0002). As indicated in the figure, among controls the LDI_FLARE_ area was not different according to presence or absence of subclinical sural nerve impairment. Similarly, the LDI_FLARE_ area was similar among cases with DSP regardless of severity. The criteria for mild neuropathy were two or more abnormal nerve conduction parameters in the lower limb (sural and peroneal nerve distributions), and moderate and severe neuropathy were defined by four and five abnormal parameters, respectively. NCS, nerve conduction study. LDI_FLARE_, laser doppler imaging flare. DSP, diabetic sensorimotor polyneuropathy.

To further explore the relationship of the LDI_FLARE_ area between healthy volunteers and subjects wtih type 1 diabetes, we examined its distribution across the different case-control categories (Figure 1). We observed a significant inverse relationship between LDI_FLARE_ area and the ordinal categories of healthy volunteers, type 1 diabetes controls without DSP divided into those with and without evidence of subclinical sural nerve injury, and type 1 diabetes cases with DSP divided into those with mild, moderate, and severe neuropathy according to the number of abnormal nerve conduction parameters (ANOVA p-value for trend, p<0.001). Compared to healthy volunteers, the type 1 diabetes controls without DSP (made up of the second and third box-and-whisker plots in Figure 1) had significantly lower LDI_FLARE_ area (p = 0.006). To determine if controls without evidence of subclinical large fibre impairment, as indicated by normal sural nerve conduction study parameters, have a different distribution of LDI_FLARE_ area than controls who have minor impairments in sural nerve conduction, we separated the distributions as indicated in the second and third box-and-whisker plots in Figure 1. Even those diabetes controls without evidence of subclinical nerve injury had lower mean LDI_FLARE_ area as compared to healthy volunteers (p = 0.03). Those controls with a single abnormal sural nerve conduction parameter had similar LDI_FLARE_ area to those with normal sural parameters (p = 0.49). However, cases with DSP had substantially lower LDI_FLARE_ area as compared to these type 1 diabetes controls without DSP (p = 0.0002). Among type 1 diabetes cases the LDI_FLARE_ area was not incrementally different in the three groups designated by increasing neuropathy severity. Despite the overall significant trend of smaller LDI_FLARE_ area with increasing nerve injury shown in Figure 1, we also noted substantial overlap in the distribution of LDI_FLARE_ area between all groups.

**Figure 2:**
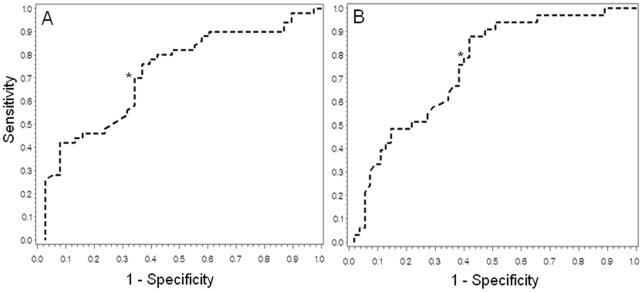
Receiver Operating Characteristic (ROC) Curves For The Identification Of Diabetic Sensorimotor Polyneuropathy (Panel A) And Subclinical Sural Nerve Impairment (Panel B) By LDI_FLARE_ Area In The 89 Subjects With Type 1 Diabetes. The ROC curve in Panel A employed as the outcome the “England criteria” for DSP. Area under the curve was 0.72 and the threshold on the curve with optimal operating characteristics (≤1.90 cm^2^ for ruling in cases, indicated by the asterisk) had a sensitivity of 66% and a specificity of 70%. The ROC curve in Panel B employed as the outcome the “sural nerve criteria” to define an earlier stage of nerve impairment that did the “England Criteria” definition for DSP. The area under the curve was 0.75and the single point on the curve with optimal operating characteristics (also ≤1.90 cm^2^ for ruling in cases, indicated by the asterisk) had a sensitivity of 79% and a specificity of 60%.

In view of the significant relationship of LDI_FLARE_ area with diabetes and neuropathy, we pursued ROC curve analysis to examine the performance of LDI to detect DSP among subjects with type 1 diabetes (Figure 2). Employing the “England criteria” for the DSP case definition the area under the ROC curve was 0.72 (Figure 2, PanelA), but the optimal threshold value for ruling in DSP of ≤1.90 cm^2^ had limited diagnostic performance (sensitivity 70% and specificity 66%). Acknowledging that the LDI_FLARE_ area represents small fibre dysfunction,we defined an alternate phenotype representing earlier nerve injury than would be defined by the “England criteria”. In this analysis we defined subclinical sural nerve impairment according to the “sural nerve criteria” and determined that the ROC curve had a higher AUC of 0.75 (Figure 2, Panel B), and an optimal threshold value (also ≤1.90 cm^2^) that had a higher sensitivity of 79%, but a lower specificity of 60% when compared to the ROC curve generated for the “England criteria” outcome.

We examined the association between LDI_FLARE_ area with the clinical and biochemical variables listed in Table 1, and report the variables that were significantly associated with LDI_FLARE_ area in Table 2. Among healthy volunteers LDI_FLARE_ area was smaller in the univariate linear regression analysis with higher weight and body mass index, higher HbA1c, and slower sural nerve conduction velocity. Among subjects with type 1 diabetes, LDI_FLARE_ area was smaller in the univariate linear regression analysis with with greater age, longer diabetes duration, higher weight and body mass index, higher systolic blood pressure, higher HbA1c, higher TCNS score and lower sural and peroneal nerve conduction parameters. In multivariate analysis, the only variable independently associated with lower LDI_FLARE_ area in both healthy volunteers and type 1 diabetes cohorts was higher HbA1c. However, higher body mass index was additionally independently associated with lower LDI_FLARE_ area in healthy volunteers.

**Table 2:** Regression Analysis Of LDI_FLARE_ Area And Baseline Demographics.

Baseline Clinical Characteristics	Healthy volunteers (n = 64)		Type 1 diabetes (n = 89)	
	β value	P value	β value	P value
Univariate models				
Female sex	−0.84	0.07	−0.39	0.15
Age (yr)	−0.01	0.36	−0.02	0.02
Diabetes Duration (yr)	–	–	−0.03	0.004
Height (m)	−1.30	0.31	−4.1	0.19
Weight (kg)	−0.02	0.05	−0.04	0.006
Body mass index (kg/m^2^)	−0.14	0.01	−0.05	0.008
Systolic blood pressure (mmHg)	−0.02	0.23	−0.03	0.003
HbA1c (%)	−2.08	0.003	−0.18	0.04
Triglycerides (mmol/L)	−0.26	0.15	−0.73	0.17
Toronto Clinical Neuropathy Score	−0.05	0.61	−0.05	0.02
Sural nerve amplitude potential (µV)	0.02	0.39	0.06	0.006
Sural nerve conduction velocity (m/s)	0.11	0.03	0.04	0.002
Peroneal nerve amplitude potential (mV)	−0.13	0.20	0.15	0.001
Peroneal nerve conduction velocity (m/s)	0.13	0.06	0.06	0.004
Multivariate model*				
Female sex	−0.16	0.77	−0.47	0.15
Age (years)	0.02	0.22	−0.02	0.08
HbA1c (%)	−2.69	0.0009	−0.21	0.04
Systolic blood pressure (mm Hg)	0.04	0.12	−0.0003	0.98
Body mass index (kg/m^2^)	−0.18	0.01	−0.03	0.39

* The multivariate model included the variables that were significant in univariate analysis in either the healthy volunteer of type 1 diabetes cohorts. Direct measures of neuropathy – which included the TCNS and the nerve conduction studies – were not considered in the multivariate analysis.

HbA1c, glycated hemoglobin A1c.

## Discussion

In a relatively large cohort of healthy volunteers and type 1 diabetes subjects with a spectrum of nerve injury, we found that there is a strong incremental association of smaller axon-mediated neurogenic vascular flare response to cutaneous heating – as measured by the LDI_FLARE_ area – from healthy volunteers, non-neuropathic type 1 diabetes subjects, and those with DSP. Despite this relationship, we found insufficient diagnostic performance of the LDI_FLARE_ area for identification of DSP according to the England clinical and electrophysiological criteria, indicated by an area under the ROC curve of 0.72, and an optimal threshold value for ruling in DSP of ≤1.90 cm^2^ associated with sensitivity of 70%. However, acknowledging limitations in the use of a later-stage outcome measure for DSP that requires the presence of clinical manifestations, we also investigated the diagnostic performance of the LDI_FLARE_ area for subclinical sural nerve impairment and found higher accuracy (area under the ROC curve, 0.75) and sensitivity (79%). Our analysis highlighted the importance of two metabolic parameters that were independently associated with LDI_FLARE_: greater glycemic exposure in diabetes subjects as well as healthy volunteers, and higher body mass index in the healthy volunteers.

There exists an urgent need to determine the early biomarkers of DSP that could be used to predict individuals at risk of subsequent clinically significant neuropathy in clinical practice, but also for the evaluation of interventions for early nerve injury in therapeutic clinical trials at the early stages when interventions are most likely to be effective. The prevailing concept of the natural history of DSP is that early nerve damage begins in small thinly myelinated Aδ and unmyelinated C-type nerve fibres [8], [9], which can be examined morphologically by quantification of intra-epidermal nerve fibre density from skin biopsy [10], or by examination of the small nerve fibres in Bowman's layer of the cornea by confocal microscopy [23]–[26]. However, evaluation of small fibre function – rather than its morphology – might offer a valid measure of the early stages of DSP, and can be examined by methods such as quantitative sensory testing and measurement of axon reflex-mediated neurogenic vasodilatation [4], [15], [16], [19], [27]–[31].

Neurogenic vasodilatation is directly related to nociceptive C-fibre function, and application of a heating stimulus activates othodromic transmission of these fibres, but also antidromic transmission along branches of the same C-fibre that supply surrounding cutaneous vessels. Additionally, there is suggestion that the flare area is also induced by a dorsal root reflex [17]. In the clinical evaluation of patients with painful DSP, the neurogenic flare was frequently observed to be impaired even in the face of normal quantitative sensory testing and normal morphology as assessed by intra-epidermal nerve fibre density [16]. In support of the attributes of the LDI_FLARE_ area as an early DSP biomarker are numerous small correlational studies [15], [16], [19], [30], [31].

The current study is among the first to evaluate the diagnostic performance of LDI_FLARE_ area in large groups of healthy volunteers and individuals with type 1 diabetes and a wide spectrum of nerve injury. Though it is cross-sectional in design, it adds to the existing literature [15], [16], [19], [30]–[32] and supports a putative role for the measurement of the LDI_FLARE_ area as a diagnostic tool for early, small nerve dysfunction in diabetes subjects. The deficiencies in its diagnostic performance shown in this analysis must be considered in the context of the outcome measure of clinical DSP according to the England criteria. The use of an outcome measure reflecting an earlier phenotype of large nerve fibre impairment in this study partially overcame deficiencies in diagnostic performance [33]. Given the absence of an established gold-standard biomarker for early nerve damage evaluated by longitudinal study, characterization of LDI_FLARE_ area as an early biomarker will be evaluated in the long-term follow-up of this cohort. This follow-up will determine if an LDI_FLARE_ area value can predict future onset of neuropathy, the ultimate indication that it represents incipient nerve impairment that is clinically relevant. Until these results are available, however, the current status of LDI_FLARE_ as a diagnostic method for the identification of DSP has substantial limitations.Whether using a DSP definition weighted toward more advanced or less advanced neuropathy severity, the sensitivity and specificity of the optimal diagnostic threshold value are insufficient as independent diagnostic tests. Rather than a diagnostic test, the role of LDI_FLARE_ may need to center on research studies designed to determine the pathogenic stages, mechansims, and the natural history of DSP.

Previous work has suggested a role for metabolic markers other than glycemic exposure – such as serum triglycerides [31] –as variables associated with the LDI_FLARE_ area. Our analysis did not identify an independent association of higher serum triglyceride with lower LDI_FLARE_ area, but did confirm the independent association with worse glycemic control as indicated by higher HbA1c levels. As a novel finding, we found an independent association of higher body mass index with lower LDI_FLARE_ area in the healthy volunteer cohort, implying that this metabolic parameter may affect small nerve function. However, we acknowledge the alternate hypothesis that increased fat mass might interfere with the technical measurement of the LDI_FLARE_ area owing to increased subcutaneous adipose tissue, and thus increase the false positive rate for the identification of neuropathy by this method. Further research into the LDI_FLARE_ area will need to reconcile the role of adiposity in the validity of this measure.

Although unique as a study of concurrent validity in DSP, we acknowledge potential limitations to the interpretation of the results in this study. First, we acknowledge that the most important clinical outcome for the identification of early neuropathy biomarkers is the future onset of DSP obtained through longitudinal study. The current cross-sectional analysis offers the first step in this longitudinal research. Second, though we had sufficient power to investigate important confounding relationships in this study, we were limited by the number of variables in multivariate analysis and could not confidently determine independent associations with all of the nerve conduction study parameters. Third, despite common features in the natural history of type 1 and type 2 diabetes, further study is required to determine the relevance of this data to type 2 diabetes. Fourth, though we see a strong association of high-normal HbA1c with lower LDI_FLARE_ area in the healthy volunteers without diabetes, we did not characterize their metabolic status by oral glucose tolerance testing. However, neither their glucose parameters or HbA1c values were consistent with a diagnosis of diabetes [34]. Finally, we did not systematically evaluate the maximum hyperemia (LDImax) in the skin immediately beneath the heating probe as performed by other investigators [31], [35].

In summary, in view of limitations in the definition of clinical neuropathy according to large fibre function, the current analysis provides substantial confirmatory evidence for the putative role of the evaluation of the neurogenic vascular flare as a biomarker of early nerve dysfunction in the natural history of DSP. Its independent association with glycemic exposure in diabetes subjects and both glycemic exposure and BMI in healthy volunteers highlights the existence of small-fibre dysfunction in the natural history of DSP.
